# Studies of La- and Pr-driven reverse distortion of FeO_6_ octahedral structure, magnetic properties and hyperfine interaction of BiFeO_3_ powder

**DOI:** 10.1039/c8ra00263k

**Published:** 2018-03-28

**Authors:** RenZheng Xiao, Tao Hu, XianBao Yuan, JianJun Zhou, XiaoQiang Ma, DeJun Fu

**Affiliations:** College of Mechanical & Power Engineering, China Three Gorges University Yichang 443002 China rzxiao@whu.edu.cn +86 0717 6397560; Hubei Key Laboratory of Hydroelectric Machinery Design & Maintenance, China Three Gorges University Yichang 443002 China; Key Laboratory of Artificial Micro- and Nano-Materials of Ministry of Education School of Physics and Technology, Wuhan University Wuhan 430072 China

## Abstract

The Bi_1−*x*−*y*_La_*x*_Pr_*y*_FeO_3_ (*x* = 0 and 0.05; *y* = 0, 0.10, 0.15 and 0.20) (BLPFO) powders were prepared using a hydrothermal method. The lattice structure of the samples was characterized by X-ray diffraction, which revealed an increase in the lattice constant of the doped samples evidencing the substitution of Bi by La and Pr ions. Raman spectroscopy was used to further analyse the structural distortion in the samples. Scanning electron microscopy was used to characterize the morphology of the samples. The atomic concentrations (%) of La and Pr elements in the samples were detected by Energy Dispersive X-ray spectroscopy. The ferromagnetism of the samples increased with the increase in La and Pr co-doping concentration as observed by vibrating sample magnetometry at room temperature. The evidence of reverse distortion of FeO_6_ octahedral structure in the La and Pr co-doped samples was revealed by the Mössbauer spectra parameters: Is, Qs, *H*, *Γ*, *χ*^2^ and area ratio (*A*_1_/*A*_2_) of two sextets.

## Abstract 2

Mössbauer spectra of the Bi_1−*x*−*y*_La*_x_*Pr*_y_*FeO_3_ (*x* = 0 and 0.05; *y* = 0, 0.10, 0.15 and 0.20) (BLPFO) powders.

## Graphical abstract

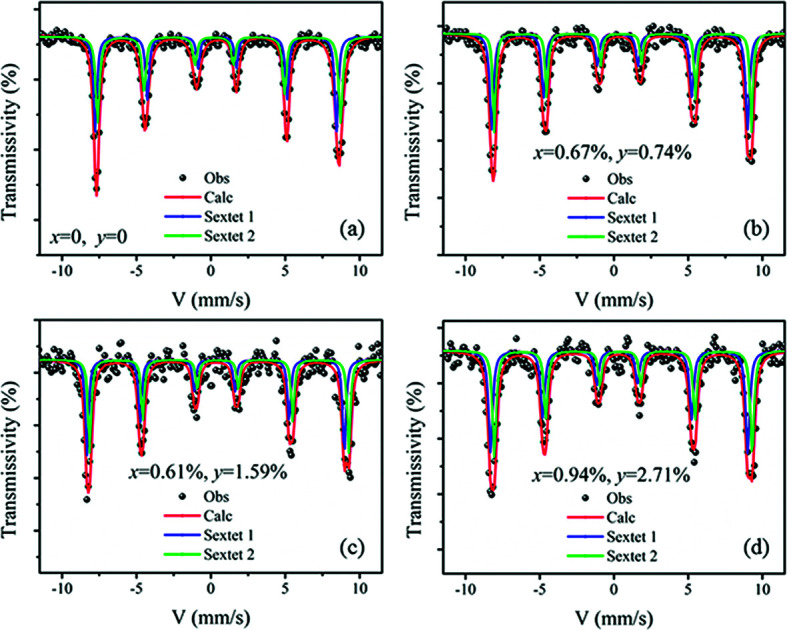

## 1. Introduction

BiFeO_3_ (BFO) has a perovskite-type structure, and it is a very typical multiferroic material, which allows the coexistence of two orders: ferroelectricity and anti-ferromagnetism with rather high ordering temperatures of ∼1143 and ∼643 K, respectively.^1,2^ In the last ten years, BFO has attracted great interest due to its potential for applications in spintronic devices, various sensors and multiple-state memories,^3–6^ which benefit by its crystal structure of a distorted rhombohedral perovskite (space group *R*3*c*)^7,8^ with the hexagonal lattice parameters of *a*_hex_ ∼ 5.58 Å and *c*_hex_ ∼ 13.90 Å.^9,10^

The Goldschmidt tolerance factor of BFO is 0.88, according the formula 
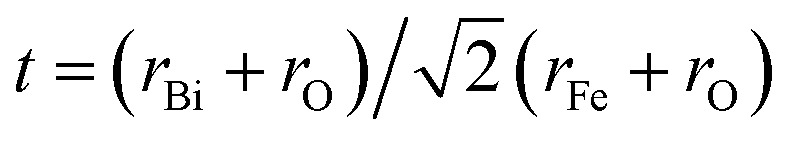
 using the ionic radii of Shannon.^5^ Therefore, the oxygen octahedra of BiFeO_3_ must be buckled to fit into a cell. Due to this, BFO possesses G-type anti-ferromagnetism or weak ferromagnetism with spontaneous magnetization in the cubic (111) plane^11^ and a long-range spin cycloid structure with a period of ∼62 nm, which is incommensurate with the lattice.^12^ The rotation angle of the oxygen octahedra is about 11–14° around the polar [111] axis, which is directly related to the Fe–O–Fe angle *θ* = 154–56°. The Fe–O–Fe angle is important because it controls both the magnetic exchange and orbital overlap between the O 2p and Fe 3d levels, which ultimately determines the local magnetic and conductivity properties.^5^ Such a distorted perovskite structure is more important to the coupling behavior between the multiferroic properties in BFO, which makes it more attractive.^13^ The magnetoelectric coupling, especially Dzyaloshinskii-Moriya interaction (DM), is connected with the exchange interaction and spin orbit coupling.^13^ The linear magnetoelectric coupling behavior between the magnetic and ferroelectric order in bulk polycrystalline BFO sample is deemed to be weak due to the cycloidal modulation of spin arrangement.^14^ The multiferroic and magnetoelectric coupling coefficients of BFO can be effectively improved by rare earth doping in A site (Bi^3+^) or B site (Fe^3+^), which is generally attributed to the distortion of the structure and leads to the destruction or modulation of the spiral spin structure.^15,16^ Dinesh Varshney *et al.* reported that the doping with rare earth Pr causes a systematic change in the structure of BFO and results in the enhancement of the magnetic behavior of the samples due to partial suppression of the spiral spin structure and stronger interaction between magnetic ions.^17–20^

Mössbauer spectrometry is a vital nuclear technique with a higher energy resolution of about 10^−8^ eV and has a unique advantage in the study of hyperfine interactions; it can be extensively used in the field of research of the hyperfine structure of BFO, which further reveals the mechanism of the modulation of the spiral spin structure. Sando *et al.* used Mössbauer and Raman spectroscopies combined with the Landau–Ginzburg theory and effective Hamiltonian calculations to show that the bulk-like cycloidal spin modulation that exists at low compressive strain is driven towards pseudo-collinear anti-ferromagnetic structure at high strain in tensile as well as compressive conditions.^21^ Landers *et al.* studied the effects of different temperatures and particle sizes on the anharmonic cycloidal spin structure in BiFeO_3_ nanoparticles.^22^ Srivastav *et al.* used ^57^Fe Mössbauer spectroscopy, which revealed that the observed enhancement in the magnetic properties of BiFeO_3_ with Pr doping is mainly due to the suppression of the modulated spiral spin structure.^15^ Kothari *et al.* explicitly showed that the magnetic properties of BiFeO_3_ with chemical substitution (Eu) are mainly due to structural distortions.^23^

In our previous study, BPFO thin film was investigated by means of magnetometry and conversion-electron Mössbauer spectroscopy (CEMS), and we had found a strong evidence of the spin cycloid destruction due to Pr doping.^24^ Based on our previous report, herein, we studied the substitution of lanthanum- and praseodymium-driven reverse distortion of FeO_6_ octahedral structure, magnetic properties and hyperfine interaction in bismuth ferrite powder. The primary objective of this study is to reveal the characteristics of FeO_6_ octahedral structure distortion in BFO caused by La and Pr co-doping and to explore the mechanism of changes in magnetic properties.

## 2. Experimental

Bi_1−*x*−*y*_La_*x*_Pr_*y*_FeO_3_ (*x* = 0 and 0.05; *y* = 0, 0.10, 0.15 and 0.20) (BLPFO) powders have been synthesized by a hydrothermal route using Fe(NO_3_)_3_·9H_2_O, Bi(NO_3_)_3_·5H_2_O, La(NO_3_)_3_·6H_2_O and Pr(NO_3_)_3_·6H_2_O as precursors, deionized water and nitric acid as solvents, and KOH as a mineralizer. The stoichiometric La/Pr/Bi/Fe ratio in the 0.2 M solution was *x* : *y* : (1 − *x* − *y*): 1, where *x* = 0 and 0.05; *y* = 0, 0.10, 0.15 and 0.20. The hydrothermal route was carried out in the reactor in an oven at a preset temperature of 200 °C for 12 hours; then, the product was washed with deionized water and dried it in the oven at 100 °C for 1 hour. Thus, the desirable samples were obtained.

The obtained powders were characterized by X-ray powder diffraction (XRD) using a Bruker AXS D8 Advance X-ray diffractometer. The Raman shift was observed by the LabRAM HR Micro Raman Spectroscopy system with a laser excitation source at 325 nm. The morphology of the samples was observed by the SIRION 200 Field Emission Gun Scanning Electron Microscope System. The concentration of elements (at%) was detected by an Energy Dispersive Spectrometer (EDS). The magnetic measurements are carried out at room temperature by vibrating sample magnetometry (VSM). The hyperfine structure of the samples was revealed at room temperature using a Wissel MS-500 Mössbauer spectrometer with a proportional counter and a ^57^Co in Rh matrix source. The initial source activity was 25.0 mCi, and the activity at the time of spectra measurement was about 21.4 mCi relative to a Fe foil with a thickness of 10 μm standard absorber with effective thickness of 10 mg Fe cm^−2^.^25,26^

## 3. Result and discussion

**Fig. 1:**
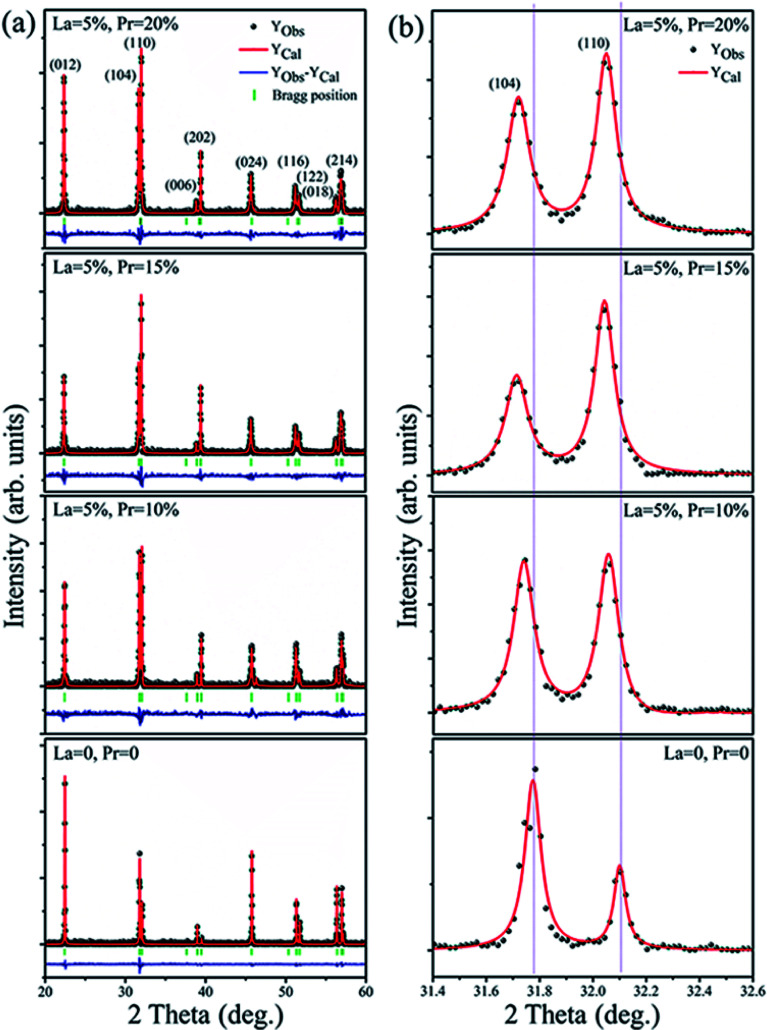
Rietveld refined XRD pattern of the Bi_1−*x*−*y*_La_*x*_Pr_*y*_FeO_3_ (*x* = 0 and 0.05; *y* = 0, 0.10, 0.15 and 0.20) powders (a); the (104) and (110) diffraction peaks in X-ray diffraction spectra of the samples (b).

The XRD pattern of Bi_1−*x*−*y*_La_*x*_Pr_*y*_FeO_3_ (*x* = 0 and 0.05; *y* = 0, 0.10, 0.15 and 0.20) powders is shown in Fig. 1a. The crystal structure of the samples has been defined as rhombohedral perovskite (*R*3*c* space group, JCPDS card no. 71-2494). No compounds aside from BLPFO (*R*3*c* space group) have been identified by XRD measurements for these samples. The Fullprof software is used for the Rietveld refinement. The simulated XRD patterns of all samples with small *R* factors are mentioned in Table 1; the simulated patterns match well with the measurements. The EDS and XRD results confirm that the increasing crystal parameters of the samples provide evidence for the substitution of Bi ions with La and Pr, which results from a slightly bigger ionic radius of La^3+^ (1.032 Å) and a smaller ionic radius of Pr^3+^ (0.99 Å) when compared with the ionic radius of Bi^3+^ (1.030 Å).^27–29^ As shown in Fig. 1b, the double split peaks corresponding to the (104) and (110) planes in BLPFO powders are distinctly observed, and they have a slight shift to the left. This suggests that the substitution of La and Pr ions in Bi_1−*x*−*y*_La_*x*_Pr_*y*_FeO_3_ enhances the lattice distortion.^30^ This distortion may have changed the bond length and bond angle of Bi–O and Fe–O covalent bonds.^31,32^

**Table 1:** Structural parameters of Bi_1−*x*−*y*_La_*x*_Pr_*y*_FeO_3_ (*x* = 0 and 0.05; *y* = 0, 0.10, 0.15 and 0.20) powders obtained by Rietveld refinement of the XRD patterns at room temperature

Bi_1−*x*−*y*_La_*x*_Pr_*y*_FeO_3_	Lattice parameters (Å), volume (Å^3^)	*R* factors	Bragg *R*-factor	Rf-factor	*χ* ^2^
*x* = 0, *y* = 0	*a* = *b* = 5.5751, *c* = 13.8578, *V* = 374.35	*R*_P_ = 9.09, *R*_wp_ = 11.71, *R*_exp_ = 8.65	2.19	1.65	1.83
*x* = 5%, *y* = 10%	*a* = *b* = 5.5785, *c* = 13.8811, *V* = 372.24	*R*_P_ = 11.46, *R*_wp_ = 13.90, *R*_exp_ = 9.93	3.78	2.49	1.96
*x* = 5%, *y* = 15%	*a* = *b* = 5.5813, *c* = 13.8762, *V* = 370.13	*R*_P_ = 12.74, *R*_wp_ = 15.57, *R*_exp_ = 10.76	4.15	3.07	2.09
*x* = 5%, *y* = 20%	*a* = *b* = 5.5776, *c* = 13.8727, *V* = 373.06	*R*_P_ = 10.83, *R*_wp_ = 12.95, *R*_exp_ = 9.97	2.82	1.94	1.69

**Fig. 2:**
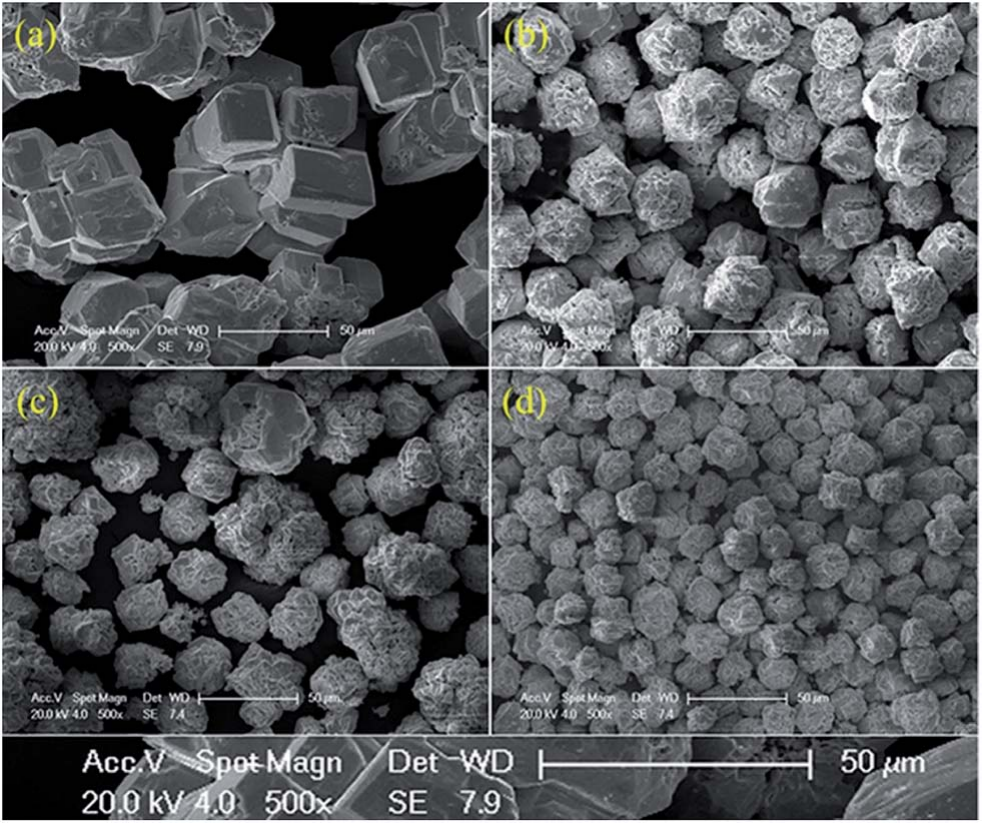
SEM morphology images of the Bi_1−*x*−*y*_La_*x*_Pr_*y*_FeO_3_ (*x* = 0 and 0.05; *y* = 0 (a), 0.10 (b), 0.15 (c) and 0.20 (d)) powders.

Fig. 2 shows the morphology of Bi_1−*x*−*y*_La_*x*_Pr_*y*_FeO_3_ (*x* = 0 and 0.05; *y* = 0, 0.10, 0.15 and 0.20) powders. The average particle sizes of Bi_1−*x*−*y*_La_*x*_Pr_*y*_FeO_3_ (*x* = 0 and 0.05; *y* = 0, 0.10, 0.15 and 0.20) powders are about 100 μm, 37 μm, 28 μm and 23 μm. It can be concluded that the particle size of BLPFO powders decreases with La and Pr co-doping, which indicates that the growth of BLPFO particles is suppressed by La and Pr co-doping. Lotey *et al.* studied Gd-doped BiFeO_3_ nanoparticles and obtained similar results.^33^ It is noteworthy that the large particle size of BLPFO powder aids in eliminating finite size effects, interparticle interactions, a random distribution of anisotropy axes and superparamagnetism.^34^

**Fig. 3:**
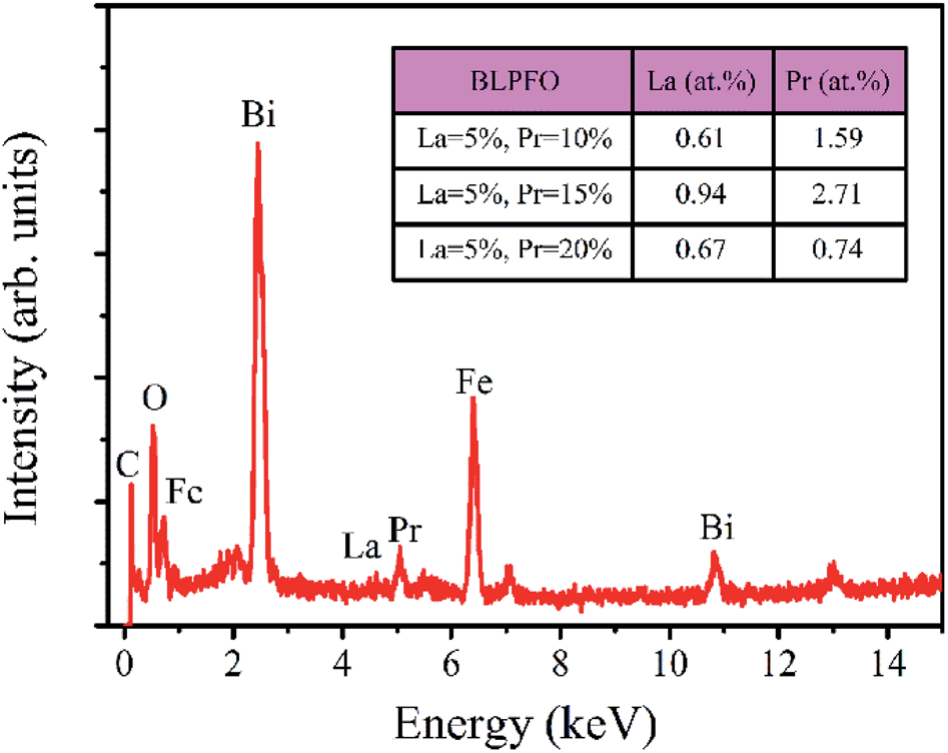
EDS spectra of Bi_0.8_La_0.05_Pr_0.15_FeO_3_ powder; the table insert in the figure shows the percentage of actual atomic concentration of La and Pr doping in Bi_1−*x*−*y*_La_*x*_Pr_*y*_FeO_3_ (*x* = 0.05; *y* = 0.10, 0.15 and 0.20) powders.

The EDS spectrum of the Bi_0.85_La_0.05_Pr_0.10_FeO_3_ powder is shown in Fig. 3. As can be seen in the insert of table in Fig. 3, the actual atomic concentrations (at%) of La and Pr doped in Bi_1−*x*−*y*_La_*x*_Pr_*y*_FeO_3_ (*x* = 0.05; *y* = 0.10, 0.15 and 0.20) powders are 0.61 at% and 1.59 at%, 0.94 at% and 2.71 at%, and 0.67 at% and 0.74 at%, respectively. The results indicate that the actual atomic concentrations of La and Pr doped in BLPFO powders are irregular and smaller than their used doses; this is owing to the solubility limitation of La and Pr elements in BLPFO powders.^33^ According to the report by Y. Zhang *et al.*,^35^ the phenomenon may be explained by that the fact that La(NO_3_)_3_ and Pr(NO_3_)_3_ transform into hydroxides under hydrothermal conditions and have different precipitation speeds and different growth characteristics. Therefore, it is necessary to reorder the samples according to the actual atomic concentration of La and Pr elements in the BLPFO powders.

**Fig. 4:**
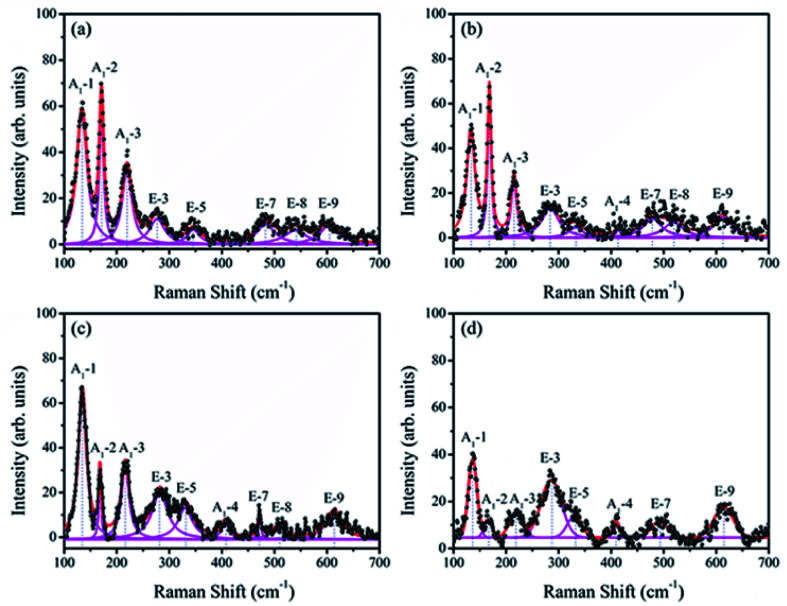
Raman scattering spectra of BiFeO_3_ (a), Bi_98.59%_La_0.67%_Pr_0.74%_FeO_3_ (b), Bi_97.80%_La_0.61%_Pr_1.59%_FeO_3_ (c) and Bi_96.35%_La_0.94%_Pr_2.71%_FeO_3_ (d) powders.

Raman spectroscopy has proved to be an excellent technique for the determination of structure more explicitly. Fig. 4 shows the Raman scattering spectra of BiFeO_3_ (a), Bi_98.59%_La_0.67%_Pr_0.74%_FeO_3_ (b), Bi_97.80%_La_0.61%_Pr_1.59%_FeO_3_ (c) and Bi_96.35%_La_0.94%_Pr_2.71%_FeO_3_ (d) powders with a measured frequency in the range of 100–700 cm^−1^. The measured spectra were simulated with individual Lorentzian components. The frequency of each Raman active mode is listed in Table 2, and each frequency was assigned based on the 13 (4A_1_ + 9E) Raman-active modes, which were predicted by the group theory in the rhombohedral (*R*3*c*) BFO crystal.^36,37^ The Raman modes observed in the low frequency region (A_1_-1, A_1_-2 and A_1_-3) were mainly related to the Bi–O vibration. The high frequency E modes located at 276, 345, 483, 538 and 605 cm^−1^ for BFO were caused by the internal vibration of FeO_6_ octahedra.^38,39^ These results were consistent with the results of previous report by Mao *et al.*^37^ As can be seen from Fig. 4, with the increasing La and Pr co-doping, the A_1_-1 mode showed a clear positive shift, and the A_1_-2 and A_1_-3 modes indicated obvious negative shifts. The E-3 and E-9 modes appeared at a distinct positive shift; the E-5 and E-8 modes indicated a negative shift. The E-7 mode first showed a negative shift and then a distinct positive shift. These results may be related to ferroelectric domain of BiFeO_3_ and can be attributed to the Fe–O bonds.^40^ In addition, the increase in the doping concentration of La and Pr resulted in significant reduction of the intensities of the A_1_-1, A_1_-2, and A_1_-3 modes, which could be attributed to the dispersion of the Bi–O bond; this also indicated that La and Pr doped into the Bi site of BFO. The A_1_-4 mode appeared in the spectrum of BLPFO samples. The E-3 and E-5 modes superposed with an increase in La and Pr co-doping. The changes in Raman modes provided an evidence for the structural distortion on Bi-sites by La and Pr co-doping, which then affected the local environment of FeO_6_ octahedra.^38,39^ The shift in the vibrational wavenumber higher than 215 cm^−1^ corresponded to the displacement of Fe and O ions in FeO_6_ octahedra as a result of distortion induced by La and Pr substitution, which could affect the Fe–O–Fe bond angle.^41^ The Raman scattering measurement results regarding the structure were consistent with XRD results revealed.

**Table 2:** Raman modes of BiFeO_3_, Bi_98.59%_La_0.67%_Pr_0.74%_FeO_3_, Bi_97.80%_La_0.61%_Pr_1.59%_FeO_3_ and Bi_96.35%_La_0.94%_Pr_2.71%_FeO_3_ powders

Samples	Assigned Raman modes (cm^−1^)								
	A_1_-1	A_1_-2	A_1_-3	E-3	E-5	A_1_-4	E-7	E-8	E-9
BiFeO_3_	133	171	219	276	345	—	483	538	605
Bi_98.59%_La_0.67%_Pr_0.74%_FeO_3_	133	169	215	283	332	413	478	518	612
Bi_97.80%_La_0.61%_Pr_1.59%_FeO_3_	134	168	216	283	330	407	472	508	613
Bi_96.35%_La_0.94%_Pr_2.71%_FeO_3_	136	166	218	285	329	409	492	—	614

**Fig. 5:**
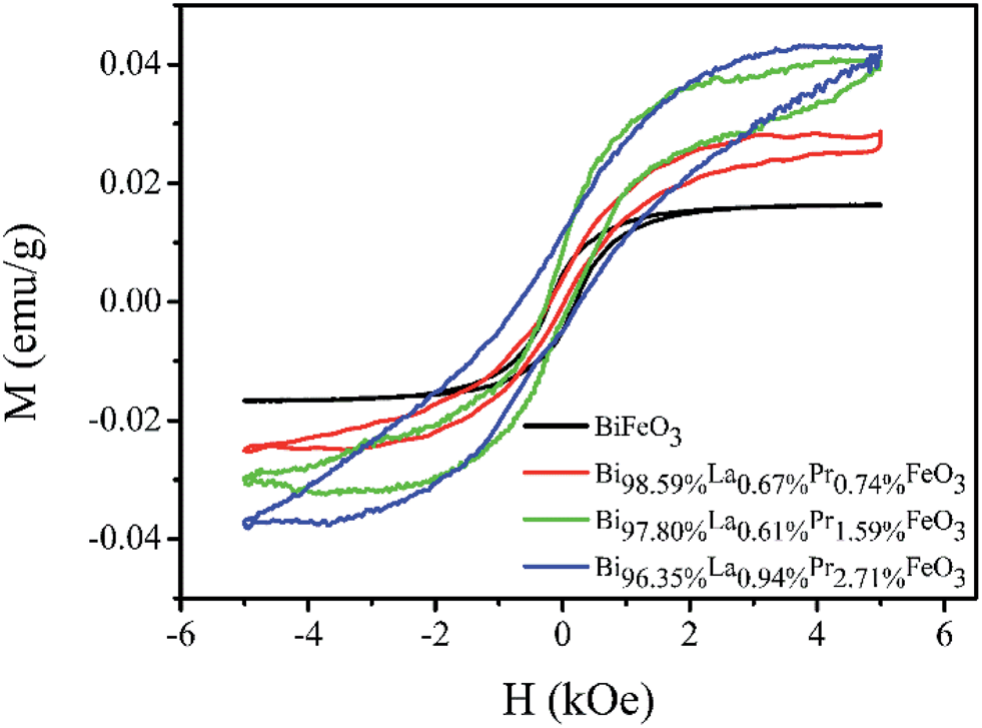
The *M*–*H* curves of the BiFeO_3_, Bi_98.59%_La_0.67%_Pr_0.74%_FeO_3_, Bi_97.80%_La_0.61%_Pr_1.59%_FeO_3_ and Bi_96.35%_La_0.94%_Pr_2.71%_FeO_3_ powders.

The weak ferromagnetism of the BiFeO_3_, Bi_98.59%_La_0.67%_Pr_0.74%_FeO_3_, Bi_97.80%_La_0.61%_Pr_1.59%_FeO_3_ and Bi_96.35%_La_0.94%_Pr_2.71%_FeO_3_ powders as a function of the external magnetic field in the range of −5–5 kOe at room temperature is shown in Fig. 5. The smaller saturation magnetization of BLPFO powders is consistent with the results reported by Lin *et al.*^42^ and Gautam *et al.*^43^ The saturation magnetization, the remnant magnetization and the coercive field of BLPFO powders are listed in Table 3. The magnetic properties of BLPFO powders enhance with La and Pr co-doping; this can be explained as a combination of several factors: (a) increase in spin canting due to the change in bond angle of Fe–O–Fe, (b) suppression of the modulated spiral spin structure, and (c) magnetic interaction between the dopants and Fe^3+^.^15^

**Table 3:** The saturation magnetization (*M*_s_), remnant magnetization (*M*_r_) and coercive field (*H*_c_) of BiFeO_3_, Bi_98.59%_La_0.67%_Pr_0.74%_FeO_3_, Bi_97.80%_La_0.61%_Pr_1.59%_FeO_3_ and Bi_96.35%_La_0.94%_Pr_2.71%_FeO_3_ powders

Samples	*M*_s_ (emu g^−1^)	*M*_r_ (emu g^−1^)	*H*_c_ (kOe)
BiFeO_3_	0.016	0.005	0.212
Bi_98.59%_La_0.67%_Pr_0.74%_FeO_3_	0.028	0.002	0.123
Bi_97.80%_La_0.61%_Pr_1.59%_FeO_3_	0.040	0.006	0.187
Bi_96.35%_La_0.94%_Pr_2.71%_FeO_3_	0.043	0.008	0.469

**Fig. 6:**
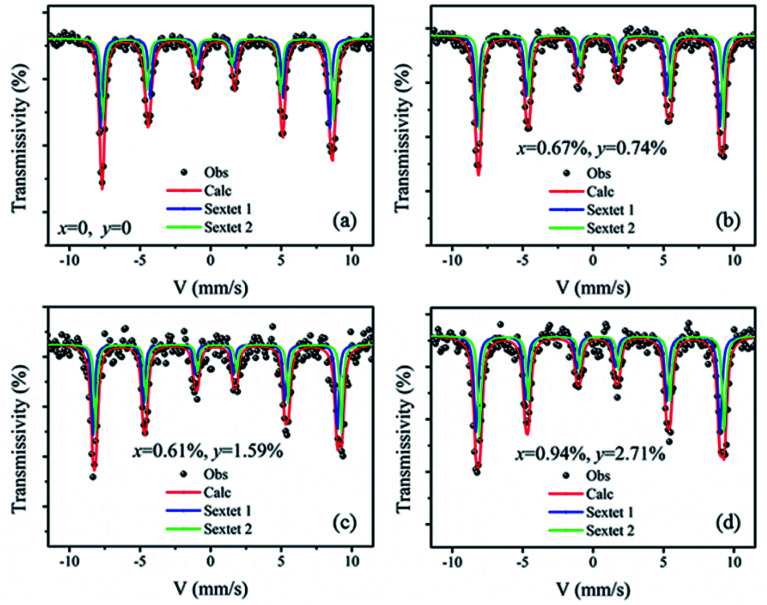
Mössbauer spectra of the BiFeO_3_ (a), Bi_98.59%_La_0.67%_Pr_0.74%_FeO_3_ (b), Bi_97.80%_La_0.61%_Pr_1.59%_FeO_3_ (c) and Bi_96.35%_La_0.94%_Pr_2.71%_FeO_3_ (d) powders at room temperature (300 K) using two sextets with a transmission integral fitting.

**Fig. 7:**
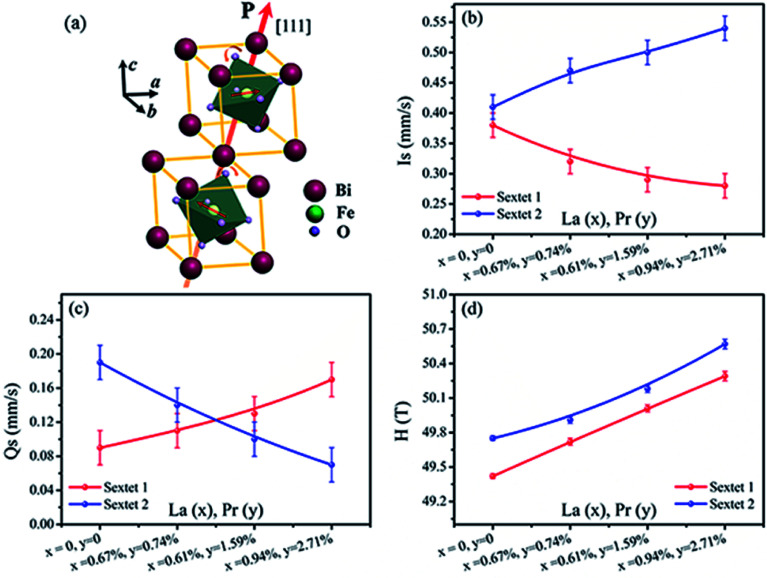
Illustration of the crystal structure of perovskite BiFeO_3_ (space group: *R*3*c*); two crystals along the [111] direction (a). The hyperfine interaction parameters Is (b), Qs (c) and *H* (d) of Mössbauer spectral lines sextet 1 and sextet 2 at room temperature dependence on the La and Pr co-doped concentration in BLPFO powders.

Fig. 6 shows the Mössbauer spectra of the BiFeO_3_, Bi_98.59%_La_0.67%_Pr_0.74%_FeO_3_, Bi_97.80%_La_0.61%_Pr_1.59%_FeO_3_ and Bi_96.35%_La_0.94%_Pr_2.71%_FeO_3_ powders at room temperature. The velocity of testing is in the range of −11–11 mm s^−1^ at the constant acceleration mode. Using two sextets with a transmission integral fitting by the MossWinn 4.0Pre program, we can obtain the Mössbauer spectra parameters: isomer shift (Is), quadrupole splitting (Qs), hyperfine magnetic field (*H*), full width at half max (*Γ*), normalized chisquare (*χ*^2^), the effective thickness (*t*_e_) and the area ratio of the subspectra (*A*_1_/*A*_2_) (shown in Table 4). As shown in Fig. 6, the black spot diagram (Obs) represents the raw data of Mössbauer spectra, the red line (Calc) is fitted spectrum, and the green and blue lines are two sextets. There is no component originating from the impure Bi_2_Fe_4_O_9_ phase (which shows doublet in the Mössbauer spectrum); the XRD results confirm the same observations. The value of *Γ* is larger than 0.5 mm s^−1^ for the Mössbauer spectra of BLPFO powders fitting by one sextet with a transmission integral (not shown here) due to the larger particles size of BLPFO powders. This is inconsistent with the real value of about 0.24 mm s^−1^ in our Mössbauer spectrum test system. The widths (FWHM) of the sextet lines for the Mössbauer spectra of BFO are *Γ*_1_ = 0.43 mm s^−1^, *Γ*_2_ = 0.54 mm s^−1^, *Γ*_3_ = 0.62 mm s^−1^, *Γ*_4_ = 0.46 mm s^−1^, *Γ*_5_ = 0.41 mm s^−1^ and *Γ*_6_ = 0.58 mm s^−1^ (not shown here). Therefore, we can conclude that *Γ*_1_ < *Γ*_6_, *Γ*_2_ > *Γ*_5_, and *Γ*_3_ > *Γ*_4_, and this phenomenon has been revealed and explained by Deepti Kothari *et al.* in detail.^23^ Such asymmetric Mössbauer spectrum of BFO is approximated by two sextets with different H values and considerably different Qs values thus concluding that Fe^3+^ ions occupy two inequivalent sites.^23,44^ The Fe–O bond lengths and Fe–O–Fe bond angles in the adjacent FeO_6_ octahedron (along the [111] direction) are different (Fig. 7a); this means that there are at least two kinds of hyperfine structures in the crystal lattice of the BLPFO powders. Therefore, at least two sextets exist in the Mössbauer spectra of BLPFO powders, which is also in accordance with the results of the studies conducted by Prado-Gonjal *et al.*^45^ In addition, the area ratio of the sextet lines is an unsatisfied ratio of 3 : 2 : 1 : 1 : 2 : 3, and this also implies the asymmetry of the Mössbauer spectra of the samples.

**Table 4:** Mössbauer spectra parameters of Bi_98.59%_La_0.67%_Pr_0.74%_FeO_3_, Bi_97.80%_La_0.61%_Pr_1.59%_FeO_3_ and Bi_96.35%_La_0.94%_Pr_2.71%_FeO_3_ powders

Bi_1−*x*−*y*_La_*x*_Pr_*y*_FeO_3_	*χ* ^2^	*t*_e_	Site	Is (mm s^−1^)	Qs (mm s^−1^)	*H* (T)	*Γ* (mm s^−1^)	*A*_1_/*A*_2_ (%)
*x* = 0, *y* = 0	1.10	7.70	Sextet 1	0.38 ± 0.01	0.09 ± 0.01	49.42 ± 0.02	0.28 ± 0.02	47.21
			Sextet 2	0.41 ± 0.01	0.19 ± 0.01	49.75 ± 0.02	0.32 ± 0.02	52.79
*x* = 0.67%, *y* = 0.74%	1.08	6.92	Sextet 1	0.32 ± 0.01	0.11 ± 0.01	49.72 ± 0.03	0.30 ± 0.02	55.73
			Sextet 2	0.47 ± 0.01	0.14 ± 0.01	49.91 ± 0.03	0.26 ± 0.02	44.27
*x* = 0.61%, *y* = 1.59%	1.26	5.83	Sextet 1	0.29 ± 0.01	0.13 ± 0.02	50.01 ± 0.03	0.26 ± 0.03	50.60
			Sextet 2	0.50 ± 0.01	0.10 ± 0.02	50.18 ± 0.03	0.24 ± 0.03	49.40
*x* = 0.94%, *y* = 2.71%	1.19	4.29	Sextet 1	0.28 ± 0.01	0.17 ± 0.02	50.29 ± 0.04	0.32 ± 0.02	50.12
			Sextet 2	0.54 ± 0.01	0.07 ± 0.02	50.57 ± 0.04	0.31 ± 0.02	49.88

Fig. 7 shows Is, Qs and *H* of sextet 1 and sextet 2 as functions of La and Pr concentration. According to the fitting results, the Qs of sextet 1 increases and the Qs of sextet 2 decreases with the increasing La and Pr co-doping concentration (Fig. 7c). This result illustrates that the lattice structure of BLPFO has been distorted by La and Pr co-doping and further reveals that the principal axis (the crystal *c* axis) of the electric field gradient (EFG) tensor of FeO_6_ octahedral structure in the nearest neighbor of the unit cell is opposite, which is formed by the lattice distortion of two positions of Fe^3+^ ions. The crystal *c* axis is the principal axis of the electric field gradient (EFG) over the period of cycloid modulation. In the case of BFO, the large contribution to EFG comes from the asymmetric O^2−^ surroundings because of the fact that the Fe^3+^ ion is not located in the center of the oxygen octahedron but is shifted along the [111] direction. Because of the DM interaction, the two Fe^3+^ magnetic moments rotate in the (111) plane resulting in spontaneous magnetization in a direction perpendicular to [111].^23^Fig. 7d shows that the H values of sextet 1 and sextet 2 increase with an increase in La and Pr co-doping concentration; this can be attributed to a change in the surroundings of Fe ion (distortion of crystal cell).^24^ This also illustrates that the destruction of the anti-ferromagnetic spin cycloid spiral structure is due to the lattice distortion by La and Pr co-doping. A possible explanation can be a connection between the increase in *H* and the spin cycloid destruction. The lattice distortion enhances the DM interaction and results in the spin cycloid destruction,^46^ which modifies the Fe–O bond lengths and Fe–O–Fe bond angles, in particular those in the Fe local environment.^28,44^ Pokatilov *et al.* studied the local states of iron ions in multiferroic Bi_1−*x*_La_*x*_FeO_3_ and claimed that the substitution of La^3+^ ions with outer electron shell 5d^1^6s^2^ for Bi^3+^ ions having an outer electron shell 6s^2^6p^3^ introduces one d electron in the samples.^47^ On the basis of this conclusion, the enhancement in hyperfine magnetic field *H* of the samples can be explained by the fact that the polarization of the s electrons in the inner closed shells enhances the Fermi contact interaction, which is caused by the exchange interaction between the s electrons and the 3d electrons of the iron ions. The result matches with the magnetic measurements. The area ratio (*A*_1_/*A*_2_) of sextet 1 and sextet 2 in Mössbauer spectra of BLPFO powders is close to 1 : 1, which suggests that the probability of finding Fe^3+^ ions in two positions of the lattice is almost the same.

Fig. 7b shows that the Is of sextet 1 and sextet 2 changed with La and Pr co-doping concentration. The Is of sextet 1 decreased and sextet 2 increased with an increase in La and Pr co-doping concentration, and it had a certain relation with the bond length and bond angle of Fe–O–Fe. This result was directly related with the density of s electron in the ^57^Fe nucleus. The change in s electron density in the valence shell and the existence of electronic shielding effect impacted the density of s electron in the ^57^Fe nucleus and thus, the Is value changed. The bond length and bond angle of Fe–O–Fe played a decisive role on the magnetic exchange interaction and the overlap of atomic orbitals between Fe and O.^48^ The overlap of atomic orbitals caused an overlap or transfer between the valence electrons of Fe^3+^ and O^2−^ ions. The overlap effect of the valence electrons had a large contribution to Is for Fe^3+^ in FeO_6_ octahedron of about 0.35 mm s^−1^,^49^ whereas the transfer effect of valence electrons had a lesser contribution to Is for Fe^3+^ in FeO_6_ octahedron of about 0.10 mm s^−1^.^50^ The distortion (tension or compression) of FeO_6_ octahedron in two different environments in the BLPFO lattice was caused by La and Pr co-doping and resulted in the Qs exhibiting a corresponding increase or decrease. The bond angle and bond length of FeO_6_ octahedron decreased or increased with the lattice distortion of BLPFO, which offered a possibility for the overlap or transfer between the valence electrons of Fe^3+^ and O^2−^ ions, and then changed the density of s electrons in the ^57^Fe nucleus.

## 4. Conclusion

In summary, we have thoroughly investigated and discussed the effects of hydrothermally substituted La and Pr ions on lattice structure, magnetic properties and hyperfine interaction in BiFeO_3_ powder. The main achievement of the performed studies is the revelation of the essential reasons for reverse distortion of FeO_6_ octahedral structure and the mechanism of magnetic enhancement. The results of XRD and Raman-scattering spectroscopy indicate the substitution of Bi by La and Pr ions, which causes the structural distortion of BiFeO_3_ and changes the local environment of FeO_6_ octahedra. The introduction of heterogeneous cations La and Pr inhibits the growth of BLPFO particles. The amounts of substitution of La and Pr ions in BiFeO_3_ is irregular as well as smaller than their used doses, which implies that La and Pr ions have different precipitation speeds and growth characteristics. We observe that La and Pr co-doping at the Bi site results in the enhancement of weak ferromagnetism of BLPFO powders. The Mössbauer spectra parameters change with the substitution of La and Pr in BiFeO_3_ powders, which clearly demonstrates that the substitution of La and Pr ions results in the reverse distortion of FeO_6_ octahedral structure. The magnetic and Mössbauer measurements conclusively and unambiguously show that the enhanced magnetic properties of BiFeO_3_ with the substitution of La and Pr are due to the destruction of the spin cycloid structure.

## Conflicts of interest

There are no conflicts to declare.

## Supplementary Material
